# The *in silico* identification of novel broad-spectrum antidotes for poisoning by organophosphate anticholinesterases

**DOI:** 10.21203/rs.3.rs-3163943/v1

**Published:** 2023-07-18

**Authors:** Sohaib Habiballah, Janice Chambers, Edward Meek, Brad Reisfeld

**Affiliations:** 1 Chemical and Biological Engineering, Colorado State University, 1370 Campus Delivery, Fort Collins, 80523-1370, CO, USA.; 2 Center for Environmental Health Sciences, College of Veterinary Medicine, Mississippi State University, 240 Wise Center Drive, Mississippi State, 39762-6100, MS, USA.; 3 Colorado School of Public Health, Colorado State University, 1612 Campus Delivery, Fort Collins, 80523-1612, CO, USA.

**Keywords:** QSAR, organophosphosphate, antidote, machine learning, virtual screening, oxime, blood-brain barrier

## Abstract

Because of their potential to cause serious adverse health effects, significant efforts have been made to develop antidotes for organophosphate (OP) anti-cholinesterases, such as nerve agents. To be optimally effective, antidotes must not only reactivate inhibited target enzymes, but also have the ability to cross the blood brain barrier (BBB). Progress has been made toward brain-penetrating acetylcholinesterase reactivators through the development of a new group of substituted phenoxyalkyl pyridinium oximes. To help in the selection and prioritization of compounds for future synthesis and testing within this class of chemicals, and to identify candidate broad-spectrum molecules, an *in silico* framework was developed to systematically generate structures and screen them for reactivation efficacy and BBB penetration potential.

## 1 Introduction

Organophosphate (OP) anticholinesterases are among the most acutely toxic synthetic chemicals known. Some OP toxicants are insecticides, but the most feared are the nerve agents, which were developed as chemical warfare agents during the second World War. The biochemical target for these toxicants is the enzyme acetylcholinesterase (AChE) which is widely distributed throughout the central and peripheral nervous systems of vertebrates. AChE is a serine hydrolase which hydrolyzes the neurotransmitter acetylcholine, thereby quickly stopping acetylcholine’s action at synapses and neuromuscular junctions. When inhibited, AChE cannot perform its vital function and excess stimulation of cholinergic pathways occurs. The actions of high exposure levels of OP toxicants can cause distress and death within minutes.

The current standard of care for OP poisoning in the United States is the use of the anticholinergic drug atropine, which antagonizes the action of excess acetylcholine at muscarinic acetylcholine receptors (the receptors most involved in the autonomic effects of OPs), and a reactivator of the inhibited AChE, pralidoxime (2-PAM), which possesses a nucleophilic oxime group that can remove the OP structure inhibiting the AChE active site and restore AChE’s action. While 2-PAM is a very good reactivator, it has the disadvantage of being only minimally able to cross the blood-brain barrier (BBB) because of the positive charge on its pyridinium ring. Therefore the AChE activity cannot be restored in the brain and the OP action can result in prolonged seizures and subsequent permanent brain damage.

A goal of our research has been to identify an oxime chemistry that would penetrate the blood-brain barrier and therefore reverse the OP inhibition in the brain and provide neuroprotection. A platform of novel substituted phenoxyalkyl pyridinium oximes (SPPOs) has provided a number of oximes [1–3] that have shown evidence of entering the brain as assessed by an increased level of AChE activity in a rat model challenged with potent OPs that serve as highly relevant surrogates of the nerve agents sarin (propan-2-yl methylphosphonofluoridate) and VX (S-2-[di(propan-2-yl)amino]ethyl O-ethyl methylphosphonothioate).

However, we have not previously attempted to utilize the structural and chemical characteristics of these SPPOs to inform the creation of new compounds that might be superior to those in our current platform. The present study was focused on the development and application of a computational framework to combinatorially generate the structures of SPPOs and screen them on their synthesizability, predicted AChE reactivation potential against a broad spectrum of OP challenges, and ability to cross the BBB.

## 2 Methods

### 2.1 Information flow

**Fig. 1:**
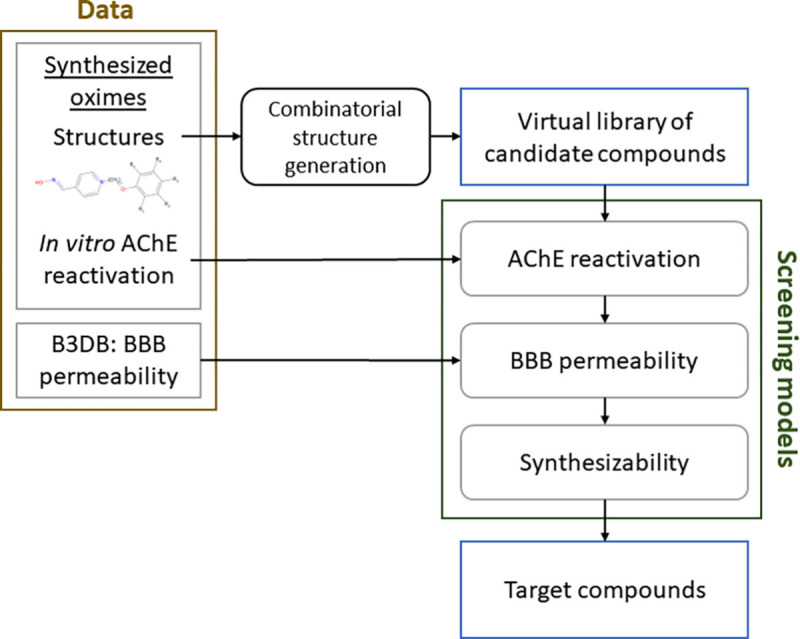
Information flow through the structure generation framework

The framework and flow of information to generate the molecular structures for the target SPPOs is shown in Figure 1. In the following sections, we detail each of these framework elements. In brief, we synthesized a number of SPPOs and quantified each compound’s ability to reactivate AChE *in vitro*. The structures of these compounds were used as the basis for generating a large virtual library of candidate molecules through the use of R-group decomposition and combinatorial substitutions. The AChE reactivation data were used to create a machine learning model to predict the reactivation potential of a SPPO given a set of structural descriptors. Through use of a large database of blood-brain barrier penetrating chemicals, we created a model to predict the level of BBB penetration of a compound based on structural descriptors. These models, along with one to evaluate chemical synthesizability, were then used to screen the virtual library of candidates and arrive at a set of target structures predicted to be synthesizable, have good reactivation potential when challenged against a spectrum of OPs, and be capable of crossing the BBB.

### 2.2 Data

#### 2.2.1 AChE reactivation

The substituted phenoxyalkyl pyridinium oximes were synthesized using the procedures detailed in Chambers et al. [4, 5]. *In vitro* acetylcholinesterase reactivation efficacy was assessed in a rat brain homogenate as described in Chambers et al. [6]. Briefly, rat brain homogenate (40 mg/ml) was incubated for 15 minutes at 37°C with an OP at a concentration that was previously determined to inhibit about 80–90% of the vehicle (ethanol) control activity. Following the inhibition phase, the OP inhibited homogenate was incubated with an oxime (100 *μ*M) or vehicle (1:1 ethanol:DMSO, vol:vol) for 30 minutes at 37°C. Each sample was then diluted (1:40) with 0.05 M Tris-HCl and assayed for acetylcholinesterase activity using a modification [7] of the Ellman method [8] with 1 mM acetylthiocholine as the substrate and 5,5’-dithiobis(nitrobenzoic acid) (DTNB) as the chromogen. The absorbance was quantified in a spectrophotometer at 412 nm. Samples containing 10 *μ*M eserine sulfate were used to correct for non-enzymatic hydrolysis. Absorbance values were then compared to uninhibited vehicle control absorbances to generate percent inhibition. Percent inhibition values from reactivated samples were then compared to the percent inhibition of the non-reactivated sample to calculate percent reactivation. At least three replicates were performed for each OP.

The aggregate dataset across studies comprised the *in vitro* AChE reactivation fraction of 84 SPPOs when challenged by each of the following four compounds: (i) Phthalimidyl isopropyl methylphosphonate (PIMP)[3]: a surrogate for the nerve agent sarin; (ii) Nitrophenyl ethyl methylphosphonate (NEMP)[3]: a surrogate for the nerve agent VX; (iii) Paraoxon (PXN)[9]: a potent metabolite of the OP insecticide parathion; and (iv) Diisopropylphosphofluoridate (DFP)[10]: a surrogate for the nerve agents sarin or soman. These data are detailed in Table S1 of the Supplementary Material.

For machine learning classification, the AChE reactivation values were converted from their continuous numerical values into discrete classes using the following system:

reactivation ≈ 0% ⇒ *No reactivation*

reactivation between 0 and 40% ⇒ *Low reactivation*

reactivation greater than 40% ⇒ *High reactivation*

The choice of 40% as a cutoff value was informed by observations of overt physical effects in test animals in our *in vivo* studies[1]. Using this classification system, the data set contained 260 *No reactivation*, 226 *Low reactivation*, and 110 *High reactivation* pairs.

#### 2.2.2 Blood-brain barrier permeability

Meng and coworkers[11] created a curated database (B3DB) containing information about the BBB permeability of hundreds of chemicals. For the purpose of this work, we selected the *regression data set* from this database, which contained logBB values for 1058 diverse molecules.

To classify molecules regarding their ability to cross the BBB, we used a threshold value of logBB, where a value above the threshold indicated a compound that would likely penetrate the BBB (*BBB+*), while that below corresponded to a compound that likely would not (*BBB−*). Several such thresholds have been used in previous studies, including 0 [12–14], 0.1 [15], and −1 [16, 17]. We selected a threshold value of logBB = 0 because a model using this value led to predictions that were consistent with observations about reactivation potential from our earlier *in vivo* studies[1]. Using this system, the resulting dataset contained 554 molecules classified as *BBB−*, with the remaining 504 labeled as *BBB+*.

### 2.3 Virtual library of candidate structures

**Fig. 2:**
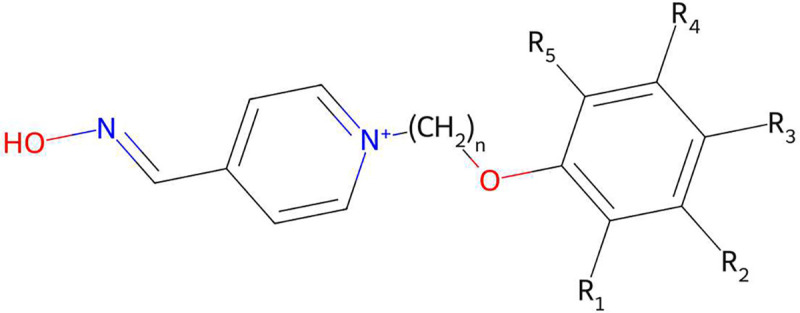
Generic structure for substituted phenoxyalkyl pyridinium oximes

Since all SPPOs shared common core scaffolds (Figure 2), we applied R-group decomposition to identify the structures and the positions of all R groups for each SPPO in the tested dataset. This procedure was facilitated by use of RDKit [18] (v2020.03.01). The resulting R-groups, R-group positions (*R*_1_-*R*_5_), and length of the linker chain, *n*, were then varied within a combinatorial analysis constrained by the following rules:

- Limit *n* to be 3, 4, or 5;
- Constrain the structure of R-groups at position *R*_3_ to those present at that location in the original SPPOs. This position had the highest variability in terms of different R groups (19 unique structures);
- Allow for R-groups to swap among positions *R*_1_, *R*_2_, *R*_4_, and *R*_5_.

These rules were put in place to assure that enumerating the set of structures was computationally feasible, while still providing a diverse set of candidate structures.

### 2.4 Screening-model development

To screen the virtual library, we developed machine learning classification models for AChE reactivation and blood-brain barrier permeability, and utilized an existing published model for synthesizability assessment.

#### 2.4.1 Acetylcholinesterase reactivation

As noted, one of the key components of our framework was a classification model to predict the reactivation of AChE against a broad spectrum of chemical challenges. Existing models detailed in the literature have utilized a variety of techniques to screen chemicals for their potential to reactivate OP-inhibited AChE, including molecular docking[19], pharmacophore modeling[20, 21], and quantum mechanical calculations[22, 23]. Though these models demonstrated good accuracy, we elected to develop a ‘fit for purpose’ model because (i) it was important to faithfully recapitulate our unique, systematic *in vitro*-based data, (ii) it was desirable to develop a self-contained ML-based predictive model more suitable to be incorporated into the overall framework, and (iii) published models focused on AChE reactivation for a single OP (e.g., tabun[23], DFP [21, 24], and methamidophos[25]), instead of accommodating multiple OP challenges.

##### Algorithm selection:

We evaluated several machine learning algorithms appropriate for classification tasks: random forest[26], naive Bayes [27], and gradient boosting [28, 29]. Focused studies indicated that both the gradient boosting and random forest algorithm were equally accurate for the current applications, but because it proved to be more computationally efficient in cases of interest, we selected the extreme gradient boosting classifier and chose the python package XGBoost[30] for the implementation.

##### Chemical descriptors:

Chemical descriptors that would comprise the model features were computed using RDkit[18] (v2020.03.01). A total of 208 descriptors were generated for each chemical (416 descriptors for each (SPPO:OP pairing) and then scaled to the range [0, 1] using min-max scaling. Descriptors with low variance across the dataset were dropped, leaving a total of 192 descriptors per chemical.

##### Feature importance assessment and optimal set selection:

A study was conducted to evaluate the influence of the number of descriptors and specific descriptors selected on the performance of the models. Subsets having fewer descriptors than the full set (FS) were called reduced sets (RS). The RS were generated iteratively and utilized the feature importance score [31] for aggregation. The performance of each model was evaluated when trained using the FS and all RS. Selecting the RS with the best F1 score resulted in the *optimal* RS, which was then used as the basis for the final models employed in the molecular screening.

##### Model training and validation:

To train and validate the models, we employed a train-validate-test technique, where 20% of the original data set was reserved for testing (testing subset), while the remaining 80% was used for training and multi-fold cross validation (training-validation subset). To help guarantee an equal representation of all classes in both subsets of data, we employed the k-mean algorithm [32] as implemented in scikit-learn [33] to cluster members of the original data set into subgroups from which samples were drawn.

Utilizing the training-validation subset, we performed a grid search to tune the model’s hyperparameters, and varied the number of trees to maximize the accuracy of the algorithm while minimizing overfitting. Models were then evaluated against the testing subset using the metrics noted above.

##### Assessing broad-spectrum reactivation:

We assessed reactivation against a total of five OP challenges: four associated with our *in vitro* studies (PIMP, NEMP, PXN, and DFP) and one based solely on model predictions (GV, 2-[dimethylamino(fluoro)phosphoryl]oxy-N,N-dimethylethanamine). We classified a SPPO as *broad spectrum* if it was predicted to have *High reactivation* potential for all five OPs. Together, these compounds represent three classes of OP nerve agents[34] (G-series, V-series, and GV-series) and a potent insecticide.

#### 2.4.2 Blood-brain barrier (BBB) permeability

Unlike for AChE reactivation, there are numerous ML-based models to predict the BBB permeability of chemicals[12–14, 16, 17, 35–37]. Though these models generally show good predictive capabilities, we chose to independently develop a model for this study because it was important to keep the same set of starting features for both the AChE reactivation and BBB permeability models so that common influential descriptors could be identified and examined. Furthermore, it was desirable to have flexibility in the selection of a cutoff value between *BBB−* and *BBB+* compounds to reflect the results of experiments related to SPPOs. The procedures used to create the classifier were the same as those described above for the AChE reactivation model.

#### 2.4.3 Synthesizability

To assess the synthesizability potential of computationally-generated SPPOs, we utilized the Retro* package [38], which is designed to find a path to synthesize a molecule from a known list of compounds and steps [39].

## 3 Results

### 3.1 Virtual library of candidate compounds

Using as input our data set containing the structures of 84 synthesized and tested SPPOs, the automated procedure described earlier led to a virtual library comprising approximately 28,000 new variations of these SPPOs.

**Fig. 3:**
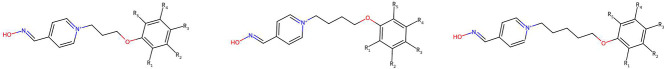
Common scaffolds among the SPPOs

**Fig. 4:**
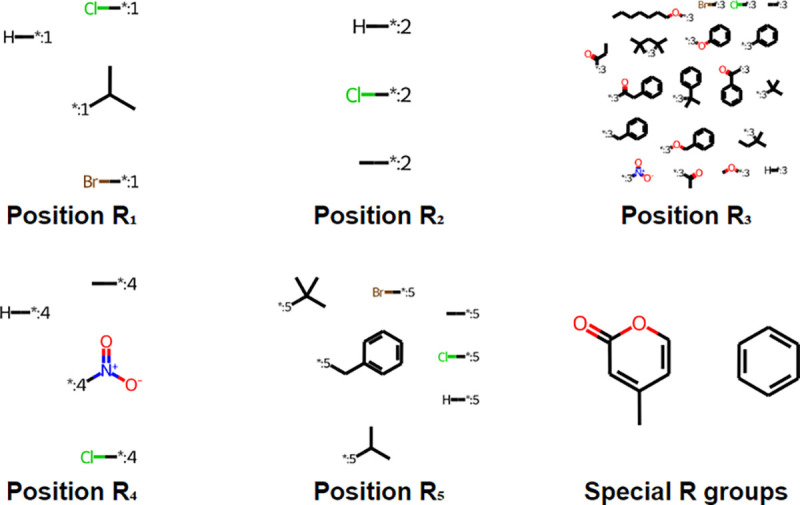
Structure and position of substituted R-groups

Result of the R-group decomposition analysis are shown in Figures 3 and 4, where the former shows the three scaffolds shared by all SPPOs in the original data set, while the latter shows all R-groups and their relevant positions on the SPPO. ‘Special R-groups’ refers to substitutions at position *R*_2_ & *R*_3_ or *R*_3_ & *R*_4_, creating a fused-ring structure.

### 3.2 AChE reactivation model

For the AChE reactivation model, the results of the feature importance assessment showed that the *optimal* RS comprised chemical descriptors from both the SPPO and the OP challenge, including topological polar surface area (TPSA), logP, quantitative estimate of drug-likeness, and number of oxime groups. The full list of 14 descriptors comprising the RS is given in Table S2 of the Supplementary Materials.

**Fig. 5:**
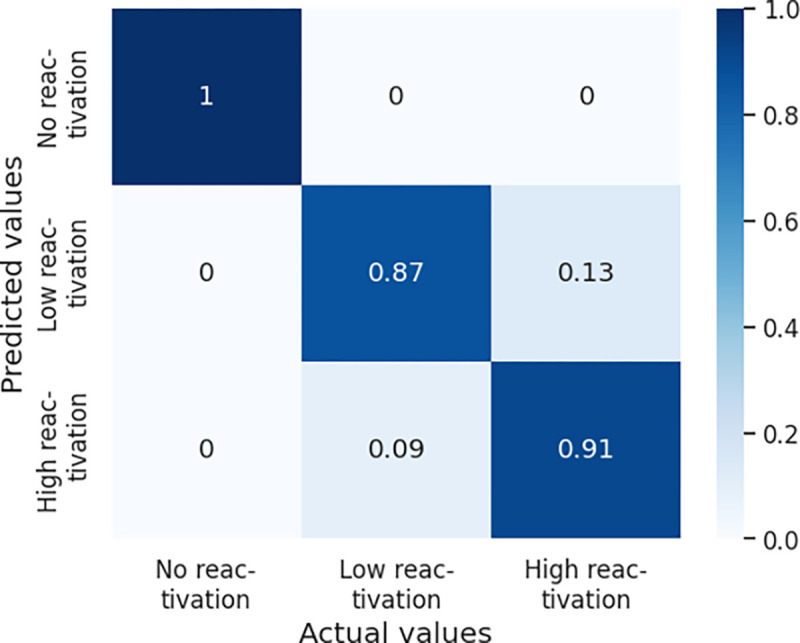
Normalized confusion matrix for the AChE reactivation model

Using the *optimal* RS and testing subset from the AChE reactivation data set, we quantified the classification performance of this model using the metrics noted earlier. The overall accuracy for the model was 92.5% and other metrics are listed in Table 1. Details of the classification performance, as represented by a normalized confusion matrix, are shown in Figure 5.

A further evaluation was conducted regarding the ability of the model to predict the AChE reactivation level when challenged by an OP that was not used in its training. In this case, the OP was phorate-oxon, the active metabolite of phorate, a phosphorodithioate insecticide. On a relatively small sample of seven SPPO:phorate-oxon pairings, the model showed an overall prediction accuracy of 72% (see Table S4 of the Supplementary Materials).

### 3.3 BBB permeability model

The results obtained from the feature importance assessment revealed that the *optimal* RS for BBB permeability prediction comprised nine chemical descriptors (see Table S3 of the Supplementary Materials), including topological polar surface area (TPSA), molecular weight, number of hydrogen bond donors, and logP.

**Fig. 6:**
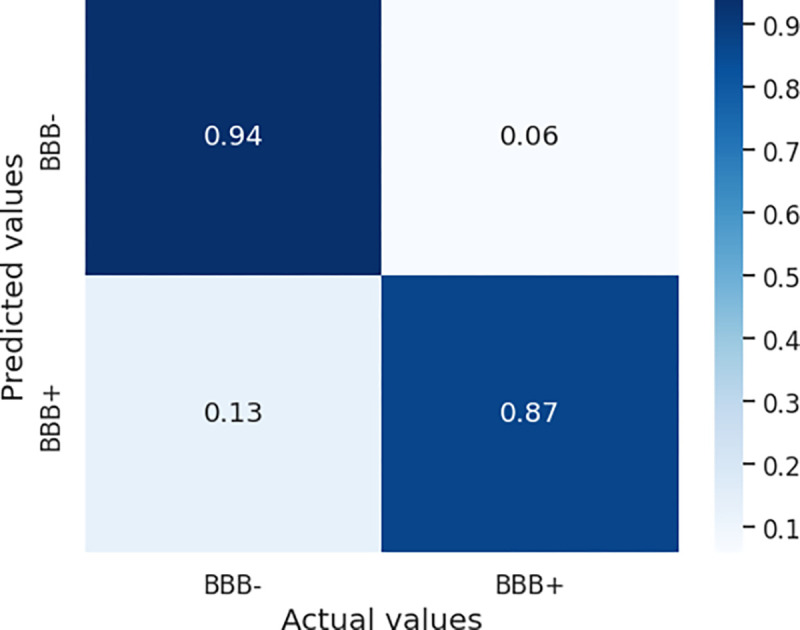
Normalized confusion matrix for BBB permeability classifier

Using the *optimal* RS and the testing subset from the blood-brain barrier permeability dataset, we evaluated the performance of this model by calculating the metrics noted earlier. This model demonstrated a good ability to discriminate *BBB+* molecules from their *BBB−* counterparts, showing a sensitivity of 89%, specificity of 94% for *BBB+*, and an overall accuracy of 90.5%. In addition, the computed F1 score for this model was 0.89. Details of the classification performance are represented by the normalized confusion matrix shown in Figure 6.

### 3.4 Target compounds

**Fig. 7:**
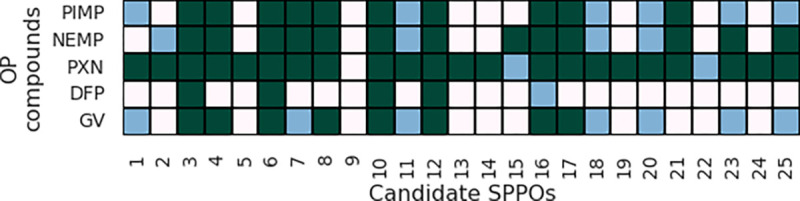
AChE reactivation level of various new SPPO:OP pairs

Of the approximately 28 thousand candidate molecules, 698 (≈2.5%) were classified as potential broad-spectrum SPPOs. A sample of the screening results is shown in Figure 7, where the intensity of the color reflects the reactivation level associated with each SPPO:OP pair. For example, candidate 3 has *High reactivation* against all tested OPs, while candidate 9 has *No reactivation* against these same challenges.

**Fig. 8:**
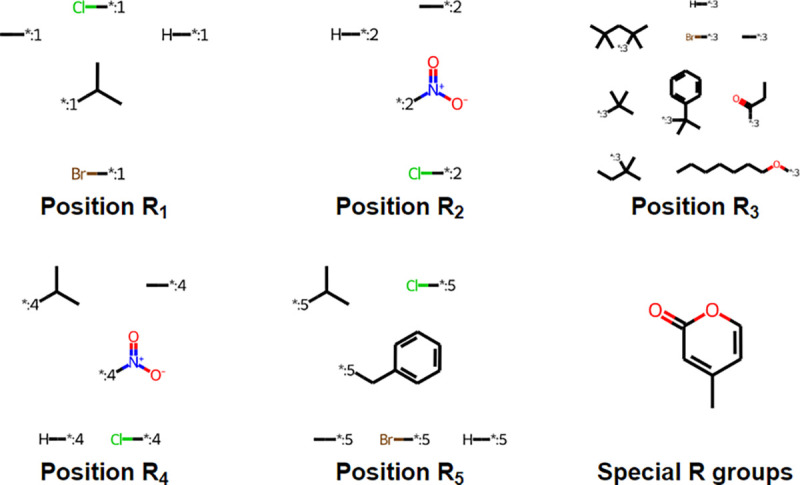
R-groups contributing to the broad-spectrum capability of the new SPPOs

To gain additional insight into the potential structural contributors to the broad-spectrum property of the new SPPOs, we applied R-group decomposition to the subset containing these compounds. The common R-groups and respective positions are shown in Figure 8.

**Fig. 9:**
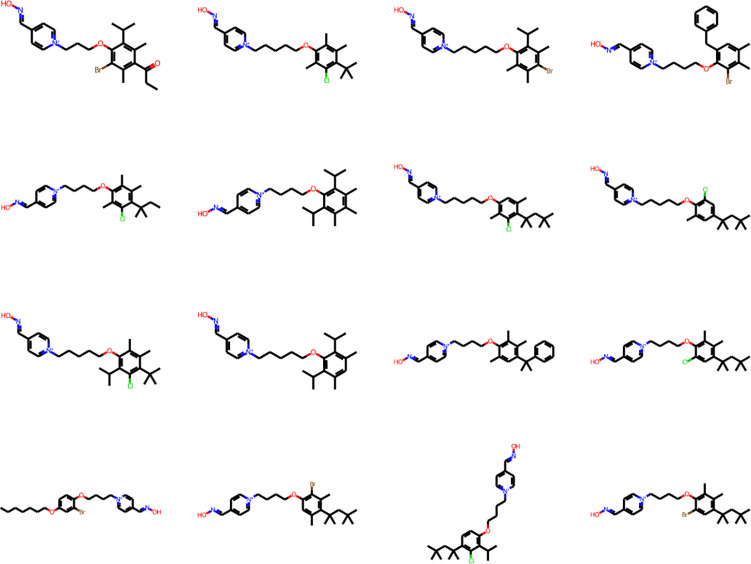
A sample of the generated structures for the target compounds

The next step of the screening revealed that 44 of the broad-spectrum candidates (6.3%) were predicted to cross the BBB. The final step, synthesizability assessment, filtered out five structures, leaving 39 SPPOs predicted to be broad-spectrum, *BBB+*, and synthesizable. A sample of these structures is shown in Figure 9. SMILES and structural information for all such structures generated in this study are provided in Tables S5 and S6 of the Supplementary Materials.

## 4 Discussion and Conclusions

In this study, we developed an *in silico* framework to help in the selection and prioritization of novel SPPOs as potential antidotes for OP nerve agent and insecticide poisoning. After combinatorially-generating a library of candidate SPPOs, we used models for AChE reactivation, BBB permeability, and synthesizability as a sequence of filters to identify suitable target compounds.

Both the AChE reactivation and BBB permeability models were developed iteratively to derive an optimal reduced feature set in each case, and models based on these feature sets were then used in the screening steps. The AChE reactivation model provided good predictive accuracy against several metrics across a set of SPPO:OP pairings, and the optimal RS included descriptors that are plausibly related to the biochemical effect, including topological polar surface area (TPSA), logP, quantitative estimate of drug-likeness, and number of oxime groups. The evaluation metrics for the BBB permeability model compared well with those of other published models [35, 40–42]. The optimal RS included descriptors, TPSA, molecular weight, number of hydrogen bond donors, and logP, that are consistent with those found in other modeling studies[37, 43], as well as with known characteristics of BBB penetrating chemicals [44].

Aside from the target set of 39 new SPPOs, the study provided information about the descriptors that may be significant in imparting the desired biological activity to the SPPOs. In particular, the most influential descriptors shared between the AChE and BBB permeability models were topological polar surface area, number of aromatic heterocycles, molecular weight, and logP. In addition, we identified common R-groups and their positions across the target compounds, and expect that this information will also be useful when selecting compounds for synthesis and testing, and in interpreting corresponding experimental results.

Despite the utility of the framework and generated structures, the approach had several limitations: (i) The underlying AChE reactivation values were based on data derived from *in vitro* studies, which do not account for various factors important for OP poisoning antidotes, such as pharmacokinetics. Adding a screening step for BBB permeability was an additional measure to begin to address this limitation. (ii) Screening for molecular synthesizability utilized a data-backed, automated retrosynthesis approach that may not reflect synthesizability in the eyes of a trained synthetic organic chemist. (iii) Though several size- and shape-related descriptors were included in the analysis, the AChE reactivation model did not include a molecular docking (or similar) component, so it cannot directly address issues related to the ligand-enzyme fit. (iv) Though the ‘best’ target SPPOs would remain the same, changing the AChE reactivation and BBB permeability classifier thresholds would influence the number of target compounds generated.

Overall, despite the limitations, the approach detailed in this study facilitated the generation of numerous new SPPOs that are predicted to be broad-spectrum antidotes for poisoning by a variety of OP anticholinesterases. We anticipate that the target compounds generated will help guide and prioritize future synthesis and testing studies in this area.

## Figures and Tables

**Table 1:** Assessment metrics for AChE reactivation model

	Sensitivity	Selectivity	F1 Score

*No reactivation*	100%	100%	1.0
*Low reactivation*	86%	96%	0.88
*High reactivation*	90%	94%	0.89

## Data Availability

The supporting *in vitro* experimental data, SMILES representations of the target compounds, Structural characteristics of the target compounds, and other information related to the models and their verification are available in the Supplementary Material.
